# Association of Low-Density Lipoprotein Receptor-Related Protein 1 and Its rs1799986 Polymorphism With Mild Cognitive Impairment in Chinese Patients With Type 2 Diabetes

**DOI:** 10.3389/fnins.2020.00743

**Published:** 2020-09-11

**Authors:** Wuyou Cao, Sai Tian, Haoqiang Zhang, Wenwen Zhu, Ke An, Jijing Shi, Yang Yuan, Shaohua Wang

**Affiliations:** ^1^Department of Endocrinology, Affiliated Zhongda Hospital of Southeast University, Nanjing, China; ^2^Medical School of Southeast University, Nanjing, China

**Keywords:** type 2 diabetes mellitus, low-density lipoprotein receptor-related protein 1, mild cognitive impairment, hyperglycemia, Alzheimer’s disease

## Abstract

**Background:**

Low-density lipoprotein receptor-related protein 1 (LRP1) is involved in cerebral glucose metabolism and amyloid-β clearance. This study aimed to investigate the pathogenetic roles of LRP1 and its rs1799986 polymorphism in mild cognitive impairment (MCI) among patients with type 2 diabetes mellitus (T2DM).

**Methods:**

A total of 166 Chinese patients with T2DM were enrolled and divided into two groups according to Montreal Cognitive Assessment (MoCA) scores. Neuropsychological tests were performed. Soluble LRP1 (sLRP1) levels were assessed using enzyme-linked immunosorbent assay, and the genotype of LRP1 rs1799986 was detected using the Sequenom method.

**Results:**

Diabetic patients with MCI (*n* = 60) exhibited significantly lower plasma sLRP1 levels (*p* = 0.033) and worse glucose control (*p* = 0.009) than the healthy cognition controls (*n* = 106). Multivariate regression analysis revealed plasma sLRP1 levels [odds ratio (OR) = 0.971, *p* = 0.005] and HbA1c (OR = 1.298, *p* = 0.003) as a risk factor for MCI in diabetic patients, in addition to insulin use and hypertension. However, there was no association between plasma sLRP1 levels and HbA1c. After adjusting for age, sex, and education level, plasma sLRP1 levels in the MCI group were negatively correlated with Stroop Color Word Test B number (*r* = −0.335, *p* = 0.011), which represents selective attention, cognitive flexibility, and processing speed. Additionally, patients with T2DM carrying the T allele of LRP1 rs1799986 showed higher Auditory Verbal Learning Test (AVLT) delayed recall scores (*p* = 0.025).

**Conclusion:**

Decreased plasma sLRP1 levels are associated with MCI, particularly with attention dysfunction, in patients with T2DM. Moreover, the T allele of LRP1 rs1799986 may decrease susceptibility to MCI. Further studies with large cohorts should be designed to elucidate the roles of LRP1 in hyperglycemia-induced cognitive decline.

## Introduction

Type 2 diabetes mellitus (T2DM) – a metabolic disorder characterized by glucose intolerance and insulin resistance – has a significant impact on human health worldwide. Furthermore, accumulating evidence has suggested that long-term hyperglycemia can lead to cognitive impairment (Cukierman et al., 2005). Patients with T2DM show a 50–60% increased risk of progressing to mild cognitive impairment (MCI) (Alarcon et al., 2005), which represents a transition stage between normal aging and dementia (Petersen, 2011). Approximately 15% of patients with MCI develop dementia annually. Moreover, T2DM is an independent risk factor for MCI and promotes progression to dementia (Cukierman et al., 2005). The etiology of T2DM-related MCI remains unclear, although it may be related to glucose and lipid metabolism disorders (Ahmad, 2013), defective insulin signaling pathways, and excessive amyloid-β (Aβ) peptide deposition in the brain (De Felice and Ferreira, 2014). Glucose homeostasis in peripheral tissues and some specific areas of the brain are both regulated through the insulin signaling pathway (Park, 2001). An impaired cerebral glucose metabolism precedes the pathological changes during early diagnosis of Alzheimer’s disease (AD) (Liu et al., 2015). Thus, identification of the factors influencing MCI in patients with T2DM is urgent to enable disease prevention and early diagnosis.

Low-density lipoprotein receptor-related protein 1 (LRP1) – a type I transmembrane protein – is a member of the low-density lipoprotein receptor (LDL-R) family (Gonias and Campana, 2014). LRP1 is a large cell surface receptor that binds to over 40 ligands, including AD-related Aβ and apolipoprotein E (ApoE) (Herz and Strickland, 2001). LRP1 is implicated in insulin signaling pathways and cerebral glucose metabolism regulation, which are closely related to cognitive function (Nakajima et al., 2014). Animal studies have demonstrated that hyperglycemia suppresses LRP1 expression and that its deficiency in neurons leads to the impairment of insulin signaling pathways and glucose uptake (Liu et al., 2015; Au et al., 2017). In addition, LRP1 plays a vital role in Aβ metabolism regulation in the central nervous system. Aβ is transported to the blood by LRP1 through the blood–brain barrier (BBB) (Tarasoff-Conway et al., 2015), and peripheral circulating Aβ is mainly transported via soluble LRP1 (sLRP1), which is a cleaved form of LRP1 circulating in the plasma (Sagare et al., 2007). In healthy individuals, approximately 70–90% of Aβ peptides bind to sLRP1, and the Aβ–sLRP1 complex cannot pass through BBB; moreover, Aβ–sLRP1 is cleared by the liver and kidneys, preventing Aβ from re-entering the brain (Tamaki et al., 2006). Aβ–sLRP1 binding is significantly reduced but plasma free Aβ40 and Aβ42 levels are significantly elevated in patients with MCI and AD. These findings suggest that plasma sLRP1 may be an early biological indicator of MCI progression to AD (Sagare et al., 2011).

The LRP1 gene is located on human chromosome 12q13-14 and contains 89 exons spanning 85 kb (Forero et al., 2006). The common LRP1 polymorphism 677C > T, a single-nucleotide polymorphism (SNP) at the position 677 of exon 3 of the LRP1 gene (database identifier dbSNP ID: rs1799986), is involved in AD and metabolic syndrome development. The LRP1 C766T polymorphism was first reported by Kang et al. (1997), and the C allele of this polymorphism is positively associated with AD susceptibility (Shinohara et al., 2017). Furthermore, C7667T (rs1799986) may be involved in amyloid deposition, particularly in ApoE ε4 carriers. However, no previous study has explored the association between the LRP1 rs1799986 polymorphism and T2DM-related MCI. A previous meta-analysis revealed a weak correlation between the LRP1 CC genotype and Aβ40 (Sanchez-Guerra et al., 2001), whereas another study showed no positive evidence of any association between LRP1 gene polymorphisms and AD risk (Pritchard et al., 2005).

Therefore, this study aimed to investigate the association of LRP1 and its rs1799986 polymorphism with MCI, particularly in different domains, among patients with T2DM. Our findings may be helpful for the screening and early diagnosis of cognitive deficits among patients with T2DM.

## Materials and Methods

### Ethics Statement

Patients with T2DM hospitalized at the Endocrinology Division of the Affiliated Zhongda Hospital of Southeast University were enrolled. The study was approved by the Research Ethics Committee of the Affiliated Zhongda Hospital of Southeast University. All participants were of Chinese Han nationality and provided signed informed consent prior to participation.

### Participants and Study Design

We included 166 patients (aged 40–80 years) who satisfied the 1999 WHO diagnostic criteria for T2DM (Alberti and Zimmet, 1998). All participants were able to cooperate and understand the procedures and had presented with T2DM for at least 3 years. The participants were divided into two groups according to Montreal Cognitive Assessment (MoCA) score: 60 patients presented with MCI (MoCA score < 26) and 106 showed healthy cognition (MoCA score ≥ 26). To reduce the effect of education level, one point was added if the participant had completed <12 years of education (Nasreddine et al., 2005). Diabetic patients with MCI met the diagnostic criteria of the National Institute on Aging Alzheimer’s Association work group (Albert et al., 2011). The exclusion criteria were as follows: (1) history of severe hypoglycemia coma, diabetic ketoacidosis, lactic acidosis, hyperosmotic sugar coma, and other acute complications of diabetes in the past 3 months; (2) history of cerebrovascular accidents confirmed by cranial imaging in the past year; (3) definite diagnosis of neurodegenerative diseases or psychological disorders, such as AD, Parkinson’s disease, or depression, within the past 2 months; (4) history of systemic diseases, such as malignant tumor, severe infection, uncontrolled hyperthyroidism, and hyperthyroidism; and (5) failure to complete the neuropsychological tests for visual and auditory discrimination defects.

### Clinical Data Collection

Patient characteristics and a detailed medical history, including age, sex, diabetes duration, education level, smoking and drinking history, hypertension (including treatment), and current diabetes treatment, were recorded through a standardized interview. Body mass index (BMI) was calculated based on weight and height [BMI = weight/height^2^ (kg/m^2^)]. Patients with systolic blood pressure of ≥140 mm Hg or diastolic blood pressure of ≥90 mmHg (Chobanian et al., 2003) or those with a history of antihypertensive drug use were considered to have hypertension. Blood samples were obtained from participants on the morning after hospital admission to collect data on glycosylated hemoglobin (HbA1c), fasting blood glucose (FBG), postprandial blood glucose (PBG), triglyceride (TG), total cholesterol (TC), low-density lipoprotein cholesterol (LDL-C), high-density lipoprotein cholesterol (HDL-C), apolipoprotein A1 (ApoA1), and apolipoprotein B (ApoB) levels. Biochemical measurements for both groups were performed in the central laboratory of the Affiliated Zhongda Hospital of Southeast University using standard laboratory techniques.

### Neuropsychological Tests

All participants were administered a series of neuropsychological tests, including the MoCA, Mini-Mental State Exam (MMSE), Digit Span Test (DST), Verbal Fluency Test (VFT), Clock Drawing Test (CDT), Logical Memory Test (LMT), Auditory Verbal Learning Test (AVLT), Trail Making Test-A and -B (TMT-A and TMT-B), and Stroop Color Word Test (SCWT). Participants’ cognitive functions, including attention, semantic memory, executive function, cognitive flexibility, processing speed, verbal and visual information, clinical dementia rating, activity of daily living scale, and self-rating depression scale, were assessed (Cai et al., 2016). An experienced neurologist from the Department of Neurology of the Affiliated Zhongda Hospital of Southeast University performed all procedures, and the participants were blinded to the study design.

### Plasma sLRP1 Levels

Blood samples (2 mL) were collected from the MCI and control groups and stored in tubes containing EDTA. The samples were then centrifuged at 100 × *g* for 15 min, and the plasma fraction was stored at −80°C. sLRP1 levels were measured using enzyme-linked immunosorbent assay kits (Jin Yibai Biological Technology, Nanjing, China), and all samples were tested on the same day to eliminate assay variance. The intra- and inter-assay coefficients of variation were <9% and 11%, respectively. The detection range of this assay was 5.0–100 pg/mL.

### Genotyping of the LRP1 rs1799986 Polymorphism

Genomic DNA was extracted from the stored protein samples using a DNA purification kit (Puregene, Gentra Systems, Minneapolis, MN, United States). The Sequenom method (CNKINGBIO, Beijing, China) was used to detect the genotype of LRP1 rs1799986. Briefly, the target regions were amplified via polymerase chain reaction (PCR), and the PCR products were processed with iPLEX^®^ Pro or iPLEX Gold reagents. A Nanodispenser RS1000 or manual pipettor was used to transfer samples from microtiter plates to a SpectroCHIP^®^ Array. The MassARRAY^®^ Analyzer was used to obtain data from the SpectroCHIP Array, which were automatically saved to the MassARRAY database. Typer software automatically analyzed the data and generated genotyping reports, which identified the SNP alleles (homozygous or heterozygous) in each sample. During MALDI-TOF, genotype calls are invoked in real time, and all measurements in multiple responses can be simultaneously visualized immediately at the end of each run. To confirm the genotyping results using Sequenom, 10% of the samples were randomly selected, and genotype analysis was conducted on 10 SNPs; there was no inconsistency.

### Statistical Analysis

Data were reported as mean and standard deviation, median (interquartile range), or number (percentage), as appropriate. Student’s *t*-test was used to compare normally distributed variables, and non-parametric Mann–Whitney *U*- and Kruskal–Wallis tests were used to compare asymmetrically distributed variables. Chi square test was used to compare qualitative variables and evaluate the genotype distribution and allele frequencies. Hardy–Weinberg equilibrium was also tested using Chi square test. Partial correlation analysis was conducted to access the association of neuropsychological test scores with clinical characteristics of participants in the two groups using Pearson’s or Spearman’s correlation. Linear regression analyses were performed to explore the factors influencing MoCA scores. Multivariate logistic regressions were employed to analyze the independent risk factors, and odds ratios (OR) were computed to estimate the relative risk. SPSS 23.0 (IBM Corp.) was used for statistical analysis, and *p*-values < 0.05 were considered statistically significant.

## Results

### Demographic Characteristics, Clinical Data, and Neuropsychological Performance

The demographic characteristics, clinical data, and neuropsychological test scores of participants are listed in Table 1. Among the 166 Chinese patients with T2DM enrolled, 60 presented with MCI and 106 with normal cognition. No significant differences were found between the two groups in terms of FBG, PBG, TG, LDL-C, HDL-C, ApoA1, and ApoB levels, smoking and drinking history, BMI, diabetes duration, and insulin use (all *p* > 0.05). The MCI group showed a higher proportion of females, lower education levels, and a larger fraction of hypertensive patients than the control group. Furthermore, HbA1c and TC levels were significantly higher in the MCI group than in the control group (*p* < 0.05). T2DM patients with MCI showed significantly lower plasma sLRP1 levels than healthy cognition controls. Neuropsychological test scores of the MCI group, except for the correct number of SCWT A Test, were significantly lower than those of the control group (*p* < 0.01).

**TABLE 1:** Demographic, clinical, and cognitive characteristics of patients with T2DM.

Characteristic	MCI group (*n* = 60)	Control group (*n* = 106)	*p*-value
Age (years)	62.20 ± 8.38	59.02 ± 8.86	0.025⁢*a
Female, n (%)	36 (60.00)	38 (35.84)	0.003⁢*c
Education level (years)	9.73 ± 3.52	11.01 ± 3.04	0.015⁢*a
Smoking status, n (%)	16 (26.67)	37 (34.91)	0.247^c^
Drinking status, n (%)	10 (16.67)	26 (24.53)	0.238^c^
BMI (kg/m^2^)	25.62 ± 3.44	24.64 ± 3.33	0.070^a^
Hypertension, n (%)	46 (76.67)	51 (48.11)	0.001⁢*c
Diabetes duration (years)	12.24 ± 6.37	10.82 ± 5.82	0.146^a^
Insulin use, n (%)	32 (53.33)	66 (62.26)	0.366^c^
HbA1c (%)	9.56 ± 2.58	8.61 ± 2.00	0.009⁢*a
FBG (mmol/L)	8.53 ± 2.89	8.05 ± 2.67	0.281^a^
PBG (mmol/L)	15.20 ± 4.13	14.40 ± 4.2	0.237^a^
Triglyceride (mmol/L)	1.74 (0.95–2.47)	1.43 (0.98–2.11)	0.141^b^
Total cholesterol (mmol/L)	4.88 ± 1.44	4.49 ± 1.11	0.049⁢*a
LDL-cholesterol (mmol/L)	2.96 ± 0.98	2.83 ± 0.83	0.364^b^
HDL-cholesterol (mmol/L)	1.21 ± 0.33	1.14 ± 0.29	0.173^a^
ApoA1 (g/L)	1.22 ± 0.30	1.12 ± 0.30	0.101^a^
ApoB (g/L)	0.90 ± 0.37	0.86 ± 0.44	0.527^a^
sLRP1 (pg/L)	54.69 (46.25–69.49)	58.43 (50.17–76.78)	0.033⁢*b
**Cognition test levels**			
MoCA	22.00 (20.25–24.00)	27.00 (26.00–28.00)	<0.001⁢*b
MMSE	27.00 (25.00–28.00)	29.00 (29.00–30.00)	<0.001⁢*b
DST	10.72 ± 1.79	12.30 ± 1.93	<0.001⁢*a
VFT	15.00 (13.00–17.00)	17.00 (15.00–21.00)	<0.001⁢*b
CDT	3.00 (2.00–4.00)	4.00 (3.00–4.00)	<0.001⁢*b
TMT-A	74.45 ± 26.04	59.33 ± 22.62	<0.001⁢*a
TMT-B	192.50 (150.50–245.00)	125.50 (98.00–164.50)	<0.001⁢*b
SCWT A time	36.73 ± 15.13	31.71 ± 13.82	0.031⁢*b
SCWT A number	49.67 ± 0.77	49.52 ± 2.99	0.374^a^
SCWT B time	58.88 ± 20.16	47.75 ± 19.28	0.001⁢*a
SCWT B number	48.00 (46.00–50.00)	50.00 (49.00–50.00)	<0.001⁢*b
SCWT C time	111.85 ± 32.86	90.49 ± 32.04	<0.001⁢*a
SCWT C number	44.60 ± 4.58	47.36 ± 3.87	<0.001⁢*b
AVLT immediate recall	15.40 ± 4.41	19.70 ± 5.15	<0.001⁢*a
AVLT delayed call	4.45 ± 2.35	6.68 ± 2.45	<0.001⁢*a
LMT	7.10 ± 3.99	11.42 ± 4.34	<0.001⁢*a

Data are presented as n (%), mean ± SD, or median (interquartile range), as appropriate. *Significant, *p* < 0.05. ^*a*^ Student’s *t*-test for the comparison of normally distributed quantitative variables between the MCI and control groups. ^*b*^ Mann–Whitney *U*-test for the comparison of asymmetrically distributed quantitative variables between the MCI and control groups. ^*c*^ Chi squire test for the comparison of qualitative variables between the MCI and control groups. MCI, mild cognitive impairment; BMI, body mass index; FBG, fasting blood glucose; HbA1c, glycosylated hemoglobin; ApoA1, apolipoprotein A1; ApoB, apolipoprotein B; LDL, low-density lipoprotein; HDL, high-density lipoprotein; sLRP1, soluble low-density lipoprotein receptor-related protein 1; MoCA, Montreal Cognitive Assessment; MMSE, Mini-Mental State Exam. DST, Digit Span Test; VFT, Verbal Fluency Test; CDT, Clock Drawing Test; TMT, Trail Making Test; SCWT, Stroop Color Word Test; AVLT, Auditory Verbal Learning Test; LMT, Logical Memory Test.

### Logistic Regression Models

Univariate logistic regression models were created, followed by multivariate regression models to explore the risk factors for T2DM-related MCI. We first included the following variables in the model: age, sex, education level, smoking and drinking history, diabetes duration, insulin use, BMI, HbA1c, FBG, PBG, HDL-C, LDL-C, TC, TG, ApoA1, ApoB, sLPR1, and the LRP1 genotype. Our results indicated that female patients with T2DM with older age, lower education level, hypertension history, higher HbA1c and ApoA1 levels, and lower plasma sLRP1 levels were highly likely to develop MCI (*p* < 0.05) (Table 2).

**TABLE 2:** Simple logistic regression model for the risk of MCI in patients with T2DM.

Risk factor	B	SE	*p*-value	OR
Age (years)	0.043	0.019	0.027*	1.043
Female, n (%)	0.987	0.332	0.003*	2.684
Education level (years)	–0.124	0.052	0.018*	0.884
Smoking status, n (%)	–0.388	0.356	0.275	0.678
Drinking status, n (%)	–0.486	0.619	0.326	1.836
BMI (kg/m^2^)	0.087	0.049	0.073	1.091
Hypertension, n (%)	1.265	0.362	0.000*	3.543
Diabetes duration (years)	0.039	0.027	0.148	1.039
Insulin use, n (%)	–0.408	0.328	0.214	0.665
HbA1c (%)	0.191	0.077	0.013*	1.211
FBG (mmol/L)	0.063	0.058	0.280	1.065
PBG (mmol/L)	0.046	0.039	0.236	1.047
Triglyceride (mmol/L)	0.201	0.111	0.070	1.222
Total cholesterol (mmol/L)	0.261	0.136	0.056	1.298
LDL-cholesterol (mmol/L)	0.167	0.184	0.362	1.182
HDL-cholesterol (mmol/L)	0.710	0.523	0.175	2.033
ApoA1 (g/L)	1.133	0.549	0.039*	3.105
ApoB (g/L)	0.240	0.384	0.532	1.271
sLRP1 (pg/L)	–0.025	0.009	0.009*	0.976
LRP1 genotype	–0.057	0.499	0.910	0.945

*Significant, *p* < 0.05. BMI, body mass index; FBG, fasting blood glucose; HbA1c, glycosylated hemoglobin; ApoA1, apolipoprotein A1; ApoB, apolipoprotein B; LDL, low-density lipoprotein; HDL, high-density lipoprotein; sLRP1, soluble low-density lipoprotein receptor-related protein 1.

Table 3 shows the independent risk factors for cognitive impairment in T2DM in the multivariate regression model. After adjustment for age, sex, and education level, multivariate regression model for the risk of MCI was optimized using a stepwise approach, with MCI as the dependent variable and other variables in Table 3 as the independent variables. The results revealed that insulin use (OR = 0.463, *p* = 0.046), hypertension (OR = 5.266, *p* = 0.000), HbA1c (OR = 1.298, *p* = 0.003), and plasma sLRP1 levels (OR = 0.971, *p* = 0.005) were associated with MCI in patients with T2MD.

**TABLE 3:** Multivariate logistic regression model for the risk of MCI in patients with T2DM.

Risk factor	B	SE	*p*-value	OR^a^
Insulin use, n (%)	0.769	0.386	0.046*	0.463
Hypertension, n (%)	–1.661	0.411	0.000*	5.266
HbA1c (%)	0.261	0.089	0.003*	1.298
sLRP1 (pg/L)	–0.029	0.010	0.005*	0.971

*Significant, *p* < 0.05. Multivariate logistic regression analysis was used to select risk factors. ^*a*^Adjusted for age, sex, and education level. MCI, mild cognitive impairment; HbA1c, glycosylated hemoglobin; sLRP1, soluble low-density lipoprotein receptor-related protein 1.

### Partial Correlations of Plasma sLRP1 Levels With Cognitive Performance and Clinical Characteristics of Patients With T2DM

The correlations of plasma sLPR1 levels with neuropsychological test scores and HbA1c levels are presented in Table 4. After adjusting for age, sex, and education level, plasma sLRP1 levels were positively correlated with MoCA (*r* = 0.177, *p* = 0.024), DST (*r* = 0.199, *p* = 0.011), and CDT (*r* = 0.182, *p* = 0.021) scores in all patients. In contrast, no significant correlations of plasma sLRP1 levels with MMSE, VFT, LMT, AVLT, SCWT, TMT-A, or TMT-B scores were noted (*p* > 0.05). In the MCI group, plasma sLRP1 levels were negatively associated with SCWT B number (*r* = −0.335, *p* = 0.011), which represents selective attention, cognitive flexibility, and processing speed. No association between plasma sLRP1 levels and HbA1c was found.

**TABLE 4:** Partial correlations of plasma sLRP1 levels with cognitive function and clinical characteristics of patients with T2DM.

	MCI group		Total	
		
	*r*	*p*-value	*r*	*p*-value
MoCA	–0.114	0.402	0.177	0.024*
MMSE	–0.215	0.111	0.082	0.302
DST	0.035	0.798	0.199	0.011*
VFT	–0.233	0.084	0.139	0.077
CDT	0.027	0.844	0.182	0.021*
TMT-A	0.031	0.823	0.117	0.139
TMT-B	0.117	0.391	0.076	0.334
SCWT A time	–0.055	0.688	–0.043	0.583
SCWT A number	0.122	0.372	–0.069	0.383
SCWT B time	0.065	0.636	–0.014	0.855
SCWT B number	–0.335	0.011*	–0.129	0.103
SCWT C time	0.139	0.305	0.015	0.848
SCWT C number	–0.309	0.777	0.046	0.560
AVLT immediate recall	–0.249	0.065	0.133	0.091
AVLT delayed call	–0.180	0.184	0.091	0.250
LMT	–0.138	0.312	0.044	0.577
HbA1c (%)	0.188	0.161	0.041	0.607

*Significant, *p* < 0.05. Partial correlation analysis adjusted for age, sex, and education level. MCI, mild cognitive impairment; sLRP1, soluble low-density lipoprotein receptor-related protein 1; MoCA, Montreal Cognitive Assessment; MMSE, Mini-Mental State Exam. DST, Digit Span Test; VFT, Verbal Fluency Test; CDT, Clock Drawing Test; TMT, Trail Making Test; SCWT, Stroop Color Word Test; AVLT, Auditory Verbal Learning Test; LMT, Logical Memory Test.

### Linear Regression Analysis

Since a positive correlation was found between MoCA scores and plasma sLRP1 levels in patients with T2DM, multiple linear regression was used to determine the risk factors that affect general cognitive performance. MoCA score was considered the dependent variable, and age, sex, education level, diabetes duration, insulin use, BMI, HbA1c, FBG, PBG, TC, TG, LDL-C, HDL-C, ApoA1, ApoB, and sLRP1 were considered the independent variables in the multiple linear regression analysis. The results (Table 5) indicated that MoCA scores were positively correlated with plasma sLRP1 levels (β = 0.181, *p* = 0.008) and negatively associated with sex (β = −0.244, *p* = 0.000), age (β = −0.146, *p* = 0.034), HbA1c level (β = −0.208, *p* = 0.002), BMI (β = −0.161, *p* = 0.028), and hypertension history (β = −2.764, *p* = 0.006).

**TABLE 5:** Linear regression analysis of factors associated with MoCA scores of patients with T2DM.

	Standardized β	95% CI		*p*-value
		
		Lower	Upper	
HbA1c (%)	–0.208	–0.500	–0.110	0.002*
sLRP1 (pg/L)	0.181	0.007	0.048	0.008*
Age (years)	–0.146	–0.106	–0.004	0.034*
Female, n (%)	–0.244	–2.523	–0.726	0.000*
BMI (kg/m^2^)	–0.161	–0.300	–0.017	0.028*
Hypertension, n (%)	–0.207	–2.380	–0.396	0.006*

*Significant, *p* < 0.05. sLRP1, soluble low-density lipoprotein receptor-related protein 1; MoCA, Montreal Cognitive Assessment; HbA1c, glycosylated hemoglobin; BMI, body mass index.

### LRP1 Genotype Distribution and Allele Frequencies Between the MCI and Control Groups

Table 6 shows LRP1 genotype distributions and allele frequencies in the MCI and control groups. The homozygous (CC) and heterozygous (CT) genotype frequencies of LRP1 rs1799986 were 88.33% and 11.67% in the MCI group and 87.74% and 11.26% in the control group, respectively; these values were consistent with the Hardy–Weinberg equilibrium for both the MCI and control groups. There were no significant differences in LRP1 genotype distributions and allele frequencies (*p* = 0.859 and *p* = 0.912, respectively) between the two groups after adjusting for age, sex, and education level.

**TABLE 6:** Distribution of the LRP1 genotype and allele frequencies between groups.

Genotype and alleles	MCI, n (%)	Control, n (%)	Crude OR (95% CI)	*p*-value	Adjusted OR^a^ (95% CI)	*p*-value
Overall	60	106				
C	113 (94.17)	199 (93.87)	1.000			
T	7 (5.83)	13 (6.13)	0.948 (0.368–2.446)	0.912		
CC	53 (88.33)	93 (87.74)	1.000		1.000	
CT	7 (11.67)	13 (11.26)	1.058 (0.398–2.817)	0.910	0.911 (0.325–2.551)	0.859
TT	0	0				

*Data are presented as n (%). Chi square test was used to compare the genotype and allele frequencies. ^*a*^ Adjusted for age, sex, and education level. MCI: Mild cognitive impairment, LRP1: Low-density lipoprotein receptor associated protein 1, OR: Odds ratio, CI: Confidence interval.*

### Comparison of Plasma sLRP1 Levels and Cognitive Performance Between Genotypic Subgroups of the MCI Group and Total Cohort

Plasma sLRP1 levels showed no significant difference between the carriers of the CC and CT genotypes in the MCI group, control group, and total patients (*p* = 0.512, *p* = 0.874, *p* = 0.895, respectively) (Table 7). Patients with MCI carrying the CT genotype showed higher AVLT delayed recall scores than those carrying the CC genotype (*p* = 0.025). In both health-condition control group and total patients, the neuropsychological tests scores identified no difference between genotypic subgroups (all *p* > 0.05).

**TABLE 7:** Comparison of plasma sLRP1 and cognitive performance plasma between genotypic subgroups.

Cognitive performance	MCI (*n* = 60)			Control (*n* = 106)			Total (*n* = 166)		
			
	CC (*n* = 53)	CT (*n* = 7)	*p*-value	CC (*n* = 93)	CT (*n* = 13)	*p*-value	CC (*n* = 146)	CT (*n* = 20)	*p*-value
sLRP1	57.9717.17	53.5610.58	0.512^a^	66.9324.39	58.0620.35	0.874^a^	63.6822.41	62.9918.64	0.895^a^
MoCA	21.553.20	22.861.35	0.291^a^	27.271.28	26.921.44	0.370^a^	25.193.51	25.502.42	0.704^a^
DST	10.771.79	10.291.80	0.502^a^	12.231.97	12.851.57	0.281^a^	11.702.03	11.952.04	0.604^a^
VFT	14.00(13.00–17.00)	14.00(13.00–14.00)	0.945^b^	18.063.95	17.153.93	0.437^a^	16.634.13	16.303.40	0.585^a^
CDT	2.881.00	3.001.16	0.780^a^	3.650.70	3.460.97	0.402^a^	3.370.90	3.301.03	0.740^a^
TMT-A	70.00(58.00–81.50)	72.00(52.00–112.00)	0.765^b^	60.0323.43	54.3116.30	0.395^a^	64.8424.63	64.4527.645	0.948^a^
TMT-B	213.7787.61	150.7150.87	0.069^a^	136.6651.72	136.8542.70	0.990^a^	164.6576.38	141.7044.89	0.192^a^
SCWT A time	36.4715.34	38.7114.37	0.716^a^	31.9414.30	30.0810.09	0.652^a^	33.5814.80	33.1012.14	0.889^a^
SCWT A number	50.00(50.00–50.00)	50.00(50.00–50.00)	0.189^b^	50.00(50.00–50.00)	50.00(50.00–50.00)	0.814^b^	50.00(50.00–50.00)	50.00(50.00–50.00)	0.416^b^
SCWT B time	58.1520.66	64.4316.07	0.444^a^	47.7020.08	48.0812.45	0.948^a^	51.4920.84	53.8015.60	0.634^a^
SCWT B number	47.512.50	48.292.56	0.445^a^	50.00(49.00–50.00)	49.00(48.00–50.00)	0.299^b^	48.532.16	47.954.12	0.328^b^
SCWT C time	111.2534.11	116.4322.47	0.698^a^	91.2732.91	84.9225.35	0.506^a^	98.5234.64	95.9528.34	0.751^a^
SCWT C number	44.604.73	44.573.46	0.986^a^	47.374.00	47.312.84	0.960^a^	46.364.47	46.353.27	0.990^a^
AVLT immediate recall	15.134.45	17.433.82	0.198^a^	19.675.12	19.925.58	0.867^a^	18.025.34	19.055.073	0.417^a^
AVLT delayed recall	4.00(2.00–6.00)	6.00(6.00–7.00)	0.025^b^*	6.602.41	7.232.74	0.389^a^	5.732.65	6.902.38	0.063^a^
LMT	6.963.98	8.144.22	0.467^a^	11.564.26	10.464.98	0.396^a^	9.894.70	9.654.75	0.331^a^

*Significant, *p* < 0.05. ^*a*^ Student’s *t*-test for the comparison of normally distributed quantitative variables between the genotypic subgroups. ^*b*^ Mann–Whitney *U*-test for the comparison of asymmetrically distributed quantitative variables between the genotypic subgroups. MCI: Mild cognitive impairment, MoCA: Montreal Cognitive Assessment, DST: Digit Span Test, VFT: Verbal Fluency Test, CDT: Clock Drawing Test, ST: Similarities Test, TMT-A: Trail Making Test-A, TMT-B: Trail Making Test-B, SCWT: Stroop Color Word Test, AVLT: Auditory Verbal Learning Test, LMT: Logical Memory Test, sLRP1: Soluble low-density lipoprotein receptor associated protein 1.

## Discussion

Our cross-sectional study examined the role of LRP1 and its rs1799986 polymorphism in T2DM-related MCI. Plasma sLRP1 levels of diabetic patients with MCI were significantly lower than those of controls. In the MCI group, the SCWT B number was negatively associated with plasma sLRP1 levels. In addition to insulin use and hypertension, increased HbAlc and decreased plasma sLRP1 levels were the risk factors for MCI in patients with T2DM. Furthermore, there were no significant differences in the distributions of LRP1 genotypes and allele frequencies between the two groups after adjusting for age, sex, and education level. However, in the MCI group, the AVLT delayed recall scores of patients carrying the CC genotype were significantly lower than those of patients carrying the CT genotype.

Long-term hyperglycemia increases the risk of cognitive dysfunction (Biessels et al., 2006). Due to the strong correlations between insulin signaling, glucose metabolism, and AD pathogenesis, LRP1 is considered to play important roles in the mechanism of T2DM-related MCI. Impairment of glucose metabolism in the brain is one of the typical pathophysiological features of AD that precedes cognitive deficits (Jack et al., 2010; Cunnane et al., 2011). An animal study has found that hyperglycemia suppressed LRP1 expression (Liu et al., 2015). Glucose transport from the blood through the BBB is mainly dependent on glucose transporters (GLUT) (Winkler et al., 2015), which are important in neuronal metabolism and for generating the energy required for cognitive function (Kandror and Pilch, 2011). Low LRP1 expression reduced GLUT3 and GLUT4 levels in neurons, subsequently impairing the insulin signaling pathway (Liu et al., 2015). Such an impairment of insulin signaling exacerbates neurodegenerative changes and synaptic loss, which ultimately leads to cognitive decline (Bosco et al., 2011; de la Monte, 2012). Therefore, LRP1 plays critical roles in regulating glucose homeostasis in the brain by controlling GLUT levels and the insulin signaling pathway. In addition, Aβ deposition in the brain is the most significant pathological characteristic of AD (Erickson et al., 2012). In the central nervous system, Aβ is mainly cleared through LRP1 across the BBB. LRP1 is a major receptor for Aβ in the liver, where the majority of plasma Aβ is cleared (Hone et al., 2003; Ghiso et al., 2004; Tamaki et al., 2006). LRP1 shows two forms, namely sLRP1 in the plasma and LRP1 on the cell surface (Zlokovic et al., 2010). In our study, we compared plasma sLRP1 levels between the control and MCI groups. Consistent with previous results (Cao and Yanping, 2018), we observed reduced plasma sLRP1 levels in the MCI group. A previous study has shown that, compared with controls, individuals with MCI who progressed to AD (MCI-AD) as well as AD patients showed, respectively, 4.9- and 3.7-fold increases in the levels of oxidized sLRP1, a form of sLRP1 that does not bind to Aβ (Zlokovic et al., 2010). Simultaneously, plasma Aβ40 and Aβ42 levels were increased in the MCI and AD groups (Zlokovic et al., 2010). Patients with MCI in our study showed higher HbA1c levels than controls, indicating worse glycemic control. High blood glucose levels suppress LRP1 expression (Liu et al., 2015), which further accelerates Aβ accumulation. Our results suggest that decreased plasma sLRP1 levels are associated with cognitive impairment in patients with T2DM. Thus, plasma sLRP1 may be an early biological indicator of MCI progression to AD.

In the partial correlation analysis, after adjustment for age, sex, and education level, plasma sLRP1 level was positively correlated with MoCA, DST, and CDT scores in all patients. MoCA was used to assess general cognitive function in multiple domains. Furthermore, DST was performed to evaluate executive function, and CDT was performed to assess verbal and visual information. In a previous study, MoCA scores were significantly lower in patients with T2DM with MCI than in healthy controls, although the scores were not significantly different between T2DM patients with and without MCI (Liu et al., 2017). Multiple linear regression was used to determine the risk factors for general cognitive performance. The results showed that MoCA scores were positively correlated with plasma sLRP1 levels and negatively correlated with sex, age, HbA1c level, BMI, and history of hypertension. These results highlighted that plasma sLPR1 levels were lower in MCI patients. Moreover, in the MCI group, the SCWT B number was negatively associated with plasma sLRP1 levels. SCWT is mainly used to evaluate selective attention, which is frequently reduced in patients with AD (Ben-David et al., 2014). A previous study showed that Aβ deposition affected specific networks, such as large-scale intrinsic connectivity networks, in patients with MCI and early AD. These networks show disrupted connectivity that affects specific cognitive functions that are impaired in AD, such as memory or attention (Koch et al., 2015). In addition, another animal study confirmed that Aβ-loaded cholinergic synapses failed to clear exogenous choline from the extracellular space in rats, marginally influencing attention functions (Parikh et al., 2014). Unfortunately, we found no association between plasma sLRP1 levels and HbA1c in the MCI group. Further studies are warranted to determine the role of LRP1 in mediating cognitive impairment in diabetes.

We performed simple and multivariate logistic regression analyses to identify risk factors associated with MCI in T2DM. The results indicated that in addition to insulin use and hypertension, HbA1c and plasma sLRP1 levels are associated with MCI in patients with T2DM. Insulin use and increased HbA1c level often indicate poor glucose control and, as mentioned previously, hyperglycemia downregulates LRP1 expression in the brain. Decreased LRP1 levels in patients with T2DM may reduce the use of brain glucose and compromise the insulin signaling pathway (Liu et al., 2015). This result may provide some evidence of the link between high blood glucose and LRP1 levels. However, another study found that patients with regular insulin use presented with better memory function after 2 and 4 months compared with patients receiving a placebo (Craft et al., 2017); this is likely because insulin administration increases brain insulin levels and improves memory and cognition in MCI or early stages of AD (Benedict et al., 2011; de la Monte, 2013), which leads to enhanced Aβ clearance from the brain (Reger et al., 2008). Furthermore, high blood pressure may increase the expression of receptors for advanced glycated end products, leading to Aβ deposition and learning impairment ability (Carnevale et al., 2016). Therefore, hypertension is a risk factor for MCI in patients with T2DM, which is consistent with previous reports (Bellew et al., 2004).

The distribution and allele frequencies of the C/T genotype of LRP1 polymorphism were compared between the two groups and no significant differences were noted. Moreover, plasma sLRP1 levels were comparable across genotypic subgroups in the MCI group. Although the LRP1 rs1799986 polymorphism is implicated in the occurrence of AD and metabolic syndrome, and the C allele is found to be positively associated with AD susceptibility (Shinohara et al., 2017), the role of the LRP1 gene related with cognitive function is still conflicting. Three meta-analysis have attempted to explore the association between the LRP1 rs1799986 polymorphism and AD susceptibility, one of which reported a weak correlation between the LRP1 CC genotype and AD (Sanchez-Guerra et al., 2001), while the other two showed no positive association between this polymorphism and AD risk (Pritchard et al., 2005) in a Chinese population (Yang et al., 2015). These differences may be attributed to several factors. First, due to the small sample size and different races, there were no TT genotypes included in our study. The LRP1 gene varies across different populations; indeed, the frequency of the T allele of exon 3 of the LRP1 gene is 22.0% and that of the C allele is 78.0% worldwide. According to a previous study, the C allele is the most common allele (Vucinic et al., 2017). Therefore, a large sample is required to evaluate the association between the LRP1 gene and MCI susceptibility. Second, the mechanism underlying T2DM-related MCI is complex and may be influenced by many genes (Jiang et al., 2017). Furthermore, environmental factors, gene mutations, and different lifestyles affect cognitive decline (McGrattan et al., 2019). Interestingly, our study showed that in the MCI group, AVLT delay recall scores of patients carrying the CC genotype were significantly lower than those of patients carrying the CT genotype, and the T allele carriers of LRP1 rs1799986 showed higher cognitive test scores. These results suggest that the T allele may be seen as a protected allele in AD, which is consistent with results of a meta-analysis published in 2017 (Kang et al., 1997). In a subgroup analysis of that meta-analysis, the T allele of LRP1 C766T was found to be associated with decreased AD susceptibility in an Asian population.

Our study is the first to investigate the association among plasma sLRP1 level, LRP1 rs1799986 polymorphism, and cognitive function in Chinese patients with T2DM. However, several limitations should be noted. First, this study was a cross-sectional study. We revealed that a decreased plasma sLRP1 level was associated with T2DM-related MCI; however, we could not clarify the causality and underlying mechanism. Second, the relatively small size and sample composition of this study led to poor matching of the control and MCI groups in terms of some baseline characteristics, such as age, sex, and education level. These confounding factors might affect cognitive performance, which limited the interpretation of our study to a certain degree. Third, since people in different ethnic populations may show different allele frequencies, the TT genotype was not detected in our study. Fourth, patients with AD or healthy volunteers without T2DM were not included; thus, the present study cannot explain whether sLRP1 levels differ between healthy individuals and patients with MCI. Therefore, further longitudinal studies with a greater sample size should be conducted to validate these findings. Finally, *APOE*, which is the strongest genetic risk factor for AD (Holtzman, 2001), is related to increased amyloid deposition in the brain (Castellano et al., 2011). Our study was unable to determine the effects of APOE on peripheral Aβ clearance. Thus, future studies should emphasize the association among APOE 4 carriers, the LRP1 gene, and plasma sLRP1 levels.

## Conclusion

Despite the aforementioned limitations, our study demonstrated that plasma sLRP1 levels are negatively correlated with cognitive performance, particularly with attention function, in patients with T2DM. Moreover, reduced plasma sLRP1 levels may increase the risk of suffering MCI in patients with T2DM. The T allele probably decreases the susceptibility to MCI. Further studies are warranted to elucidate whether LRP1 mediates cognitive decline caused by hyperglycemia, and the association between the LRP1 rs1799986 polymorphism and MCI in T2DM should be validated in future large population studies.

## Data Availability Statement

Database of Single Nucleotide Polymorphisms (dbSNP). Bethesda (MD): National Center for Biotechnology Information, National Library of Medicine. dbSNP accession: {ss2137544094} (dbSNP Build ID: {build155}). Available from: http://www.ncbi.nlm.nih.gov/SNP/.

## Ethics Statement

The studies involving human participants were reviewed and approved by the Affiliated Zhongda Hospital of Southeast University Research Ethics Committee. Patients/participants provided their written informed consent to participate in the study.

## Author Contributions

SW and YY conceived the idea and revised the manuscript. WC designed the study and wrote the manuscript. KA, HZ, JS, and WZ collected the data. ST participated in the data analysis and interpretation. All authors read and approved the submitted version of this manuscript.

## Conflict of Interest

The authors declare that the research was conducted in the absence of any commercial or financial relationships that could be construed as a potential conflict of interest.
